# Controlling doping efficiency in organic semiconductors by tuning short-range overscreening

**DOI:** 10.1038/s41467-023-36748-x

**Published:** 2023-03-13

**Authors:** Jonas Armleder, Tobias Neumann, Franz Symalla, Timo Strunk, Jorge Enrique Olivares Peña, Wolfgang Wenzel, Artem Fediai

**Affiliations:** 1 Institute of Nanotechnology, Karlsruhe Institute of Technology, Eggenstein-Leopoldshafen, Germany; 2 Nanomatch GmbH, Griesbachstraße 5, 76185 Karlsruhe, Germany

**Keywords:** Electronic devices, Electronic properties and materials, Theory and computation, Molecular electronics

## Abstract

Conductivity doping has emerged as an indispensable method to overcome the inherently low conductivity of amorphous organic semiconductors, which presents a great challenge in organic electronics applications. While tuning ionization potential and electron affinity of dopant and matrix is a common approach to control the doping efficiency, many other effects also play an important role. Here, we show that the quadrupole moment of the dopant anion in conjunction with the mutual near-field host-dopant orientation have a crucial impact on the conductivity. In particular, a large positive quadrupole moment of a dopant leads to an overscreening in host-dopant integer charge transfer complexes. Exploitation of this effect may enhance the conductivity by several orders of magnitude. This finding paves the way to a computer-aided systematic and efficient design of highly conducting amorphous small molecule doped organic semiconductors.

## Abstract (web summary)

Doping is widely adopted to make organic semiconductors more conductive, yet the impact of molecular electronic properties on doping performance is still not fully understood. Armleder et al. compute host-dopant interactions and show that a short-range overscreening effect strongly affects conductivity.

## Introduction

In the past decades, organic semiconductors (OS) have made their way into many electronic applications on an industrial scale, especially organic light-emitting diodes (OLEDs)^1^, and are experiencing increasing interest in emerging technologies, such as organic solar cells (OSC)^2^ and organic field-effect transistors^3^. One of the challenges of this class of materials is their low intrinsic mobility^4,5^, orders of magnitude below that of the best inorganic semiconductors. Since the first reports by Blochwitz et al. and Yamamori et al.^6,7^, doping has evolved into a promising approach to enhance electrical conductivity. As doped OSC layers can further be used to eliminate injection barriers at electrodes, doping is by now an indispensable method in boosting the performance of OLEDs and other organic electronic devices^8–13^.

On a microscopic level, doping of OS is a two-step-process^14^: 1. A charge transfer (CT) between host and dopant molecules leads to activation of the host-dopant pair. (In this study, we will focus on integer CT (ICT) and not consider fractional CT studied elsewhere^15,16^. The Coulomb complex of the oppositely charged host and dopant, in this case, is referred to as integer CT complex (ICTC)). 2. Dissociation of the charge carrier on the host matrix from the charged dopant generates a free charge to contribute to charge transport. We will assume p-doping only for the sake of conciseness. A key quantity in both steps is the electrostatic binding energy *V*_C_ between host-dopant pairs in the ICTC (ICTC binding energy). On the one hand, this energy stabilizes the ICTC, i.e., aids dopant ionization, but on the other hand, poses a large barrier to be overcome by a charge carrier to contribute to the conductivity^17–19^.

The correlation of *V*_C_ with doping efficiency has been discussed in^14,17,18,20–23^ at various levels of the theory. However, existing work often uses a simplified picture by considering *V*_C_ to be a fixed number for some particular host-dopant combination^21,22^ or a distance-dependent function of *V*_C_(*r*) in the case of crystalline materials^24,25^. These approaches do not take into account the amorphous nature of disordered OS that induces an additional dependence of *V*_C_(*r*) on the unique relative orientations between each host-dopant pair. Further, the polarization effects of the environment of host–dopant complexes are often ignored^21^. Existing approaches, therefore, lack the ability to formulate design criteria and conduct reliable in silico searches for efficient dopants.

In this work, we go beyond existing approaches by developing a microscopic model to compute host–dopant interactions and integrating this explicit computation of *V*_C_(*r*) into a multiscale ab initio model previously applied to only intrinsic OS^5,26–28^. In digital twins of OSC thin films, we compute host–dopant interactions *V*_C_(*r*) for pairs at various distances and relative orientations. We explicitly take into account the electrostatic response of the unique environment of each pair with quantum accuracy by extending a recent method for intrinsic semiconductors^19,29–31^. This model, therefore, takes into account the three essential factors to reliably evaluate doping efficiency in amorphous OS: distance dependence of *V*_C_(*r*), the disorder of *V*_C_(*r*) induced by various relative orientations, and the effect of the polarization effects in the environment of ICT states. The resulting distributions are used to parametrize a kinetic Monte Carlo (kMC) model^32^ to compute the conductivity of the doped OSCs, thereby bridging the gap between fundamental chemistry and functionality on a device level. We applied this workflow to five prototypical host-dopant combinations and identified relative molecular orientations as the cause for overscreening effects, i.e., deviation from the screened Coulomb law of two oppositely charged point charges, at short host-dopant distances. We systematically investigated the impact of the microelectrostatics of dopants on doping efficiency, suggesting the potential to tune the conductivity of up to two orders of magnitude by molecular design. Ultimately, we validate our model against experimental data. We show that the width of the *V*_C_(*r*) distribution reduces conductivity and therefore plays a similar role as energy disorder in intrinsic OSC.

## Results

### Short-range effects in integer charge-transfer complexes

**Fig. 1:**
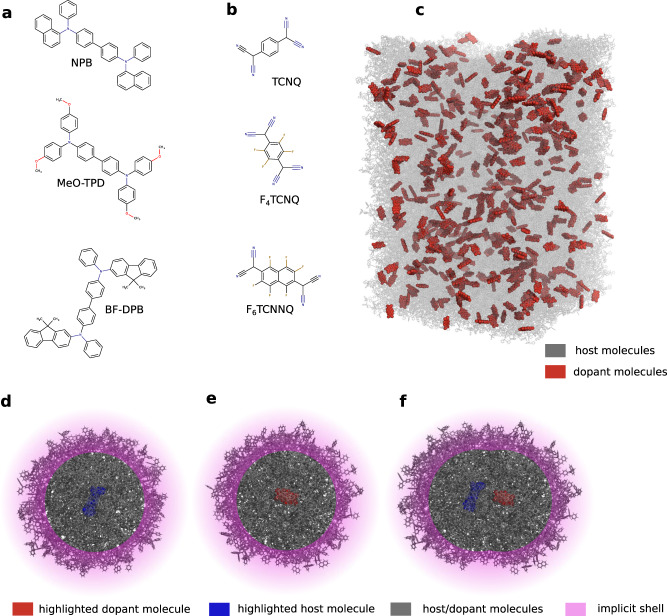
Doped organic semiconductor materials and morphologies. **a** Host molecules. **b** Dopants TCNQ, F_4_TCNQ, and F_6_TCNNQ (top to bottom) with progressively increasing quadrupole moments component *Q*_*zz*_ (31.6 $e{a}_{0}^{2}$, 36.8 $e{a}_{0}^{2}$, 51.0 $e{a}_{0}^{2}$, respectively). **c** Distribution of dopants in a host matrix, exemplified for NPB:F_6_TCNNQ (5 mol%), dopants shown in red. **d**–**f** CT binding energy is computed via different charged states in comparison to the neutral state of a host–dopant pair embedded in the explicit polarization shell: IP is computed via a charged host (blue) and neutral dopant, EA via charged dopant (red) and neutral host. *E*_CT_ is computed via the CT state.

To understand the factors influencing the ICTC binding energy in disordered OS, *V*_C_, we first conduct a detailed analysis of a prototypical material combination NPB:F_6_TCNNQ (5 mol%). The molecular structures are depicted in Fig. 1a, b, full names of the molecules are provided in Supplementary Table 1. Specifically, we computed *V*_C_ using the quantum embedding method “QuantumPatch”^29–31,33^ for 30 host-dopant pairs with different center-of-geometry distances. To this end, we compute the energy of each embedded host–dopant pair in the neutral and three charged states, as exemplified in Fig. 1d–f, to obtain host IP (IP_host_), dopant EA (EA_dop_), and the energy of the ICTC binding energy. The ICTC binding energy is then computed according to Eq. (8). Details can be found in Section “Estimation of the Coulomb binding energy *V*_C_ distribution”.

**Fig. 2:**
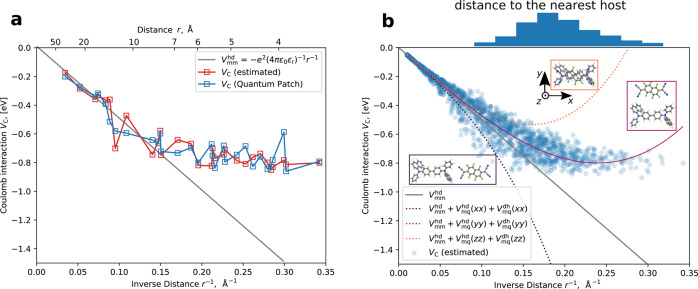
Short-range overscreening effect. **a** Dependence of the ICT state binding energy *V*_C_ between charged host-dopant pairs on the inverse distance between them, *r*^−1^, in NPB:F_6_TCNNQ (5%), computed with quantum embedding (QuantumPatch, (blue)) method and estimated via a multipole representation (red), in comparison with classical Coulomb interaction between two monopoles in polarizable medium with dielectric permittivity *ϵ*_r_ = 2.7) (gray line). For short distances, *V*_C_ of our proposed model is weaker than classical monopole–monopole interaction. **b** *V*_C_(*r*^−1^) estimated for 10,000 pairs indicates a distance-dependent disorder of *V*_C_. On top: distribution of the distances from a dopant to the nearest host molecules. Auxiliary lines are sums of monopole–monopole and monopole–quadrupole interactions in three orthogonal host–dopant orientations, as shown in the insets. The orientation of the host and dopants relative to the coordinate system is also shown in Supplementary Fig. 1.

The dependence of *V*_C_ on the inverse distance in comparison with the classical Coulomb interaction between two monopoles in a polarizable medium with dielectric permittivity *ϵ*_r_ = 2.7), ${V}_{{{{{{{{\rm{mm}}}}}}}}}^{{{{{{{{\rm{hd}}}}}}}}}$, is depicted in Fig. 2a. At short distances (*r* < 6.7 Å, *r*^−1^ > 0.15 Å^−^^1^), *V*_C_ deviates from the classical Coulomb interaction. Notably, it is almost distance-independent for *r* < 6.7 Å, leading to a deviation of 0.7–0.9 eV from the classical Coulomb interaction at *r* = 3.3 Å. After ionization of a host–dopant pair, the generated hole, therefore, moves in a flat electrostatics potential (until the *r* ≈ 6.7 Å) rather than being captured into a deep Coulomb trap next to the host cation. We refer to this effect as short-range overscreening.

In order to compute statistically significant distributions of *V*_C_ (i.e., on multiple hundred of host–dopant pairs), we developed an estimator based on a multipole representation of the dopant and partial charge representation of the host molecule (see “Estimation of the Coulomb binding energy *V*_C_ distribution” for details). While the comparison between *V*_C_ computed with the estimator and the “QuantumPatch” approach in 2a (blue data and red data, respectively) show notable deviations for individual pairs, these deviations remain within the fluctuation within either method for pairs with similar distances. The distribution of *V*_C_, computed via the estimator for approximated 10,000 host–dopant pairs in the morphology, uniformly distributed over 1/*r*, is depicted in 2b.

To qualitatively understand the distribution of *V*_C_ across host–dopant distances *r*, we modeled the electrostatic properties of host and dopant molecules via monopole and quadrupole moments. In this case, the Coulomb interaction between host cations and dopant anions can be approximated as:

1 $${V}_{{{{{{{{\rm{C}}}}}}}}}\,({{\mbox{multipoles-multipoles}}})\,={V}_{{{{{{{{\rm{mm}}}}}}}}}^{{{{{{{{\rm{hd}}}}}}}}}+{V}_{{{{{{{{\rm{mQ}}}}}}}}}^{{{{{{{{\rm{hd}}}}}}}}}+{V}_{{{{{{{{\rm{mQ}}}}}}}}}^{{{{{{{{\rm{dh}}}}}}}}}$$

where

2 $${V}_{{{{{{{{\rm{mm}}}}}}}}}^{{{{{{{{\rm{hd}}}}}}}}}=-\frac{{e}^{2}}{4\pi {\epsilon }_{0}{\epsilon }_{{{{{{{{\rm{r}}}}}}}}}r}$$

is the monopole-monopole interaction,

3 $${V}_{{{{{{{{\rm{mQ}}}}}}}}}^{{{{{{{{\rm{hd}}}}}}}}}=+ \frac{e}{8\pi {\epsilon }_{0}{\epsilon }_{{{{{{{{\rm{r}}}}}}}}}}\frac{{{{{{{{\bf{r}}}}}}}}{{{{{{{{\bf{Q}}}}}}}}}^{{{{{{{{\rm{d}}}}}}}}}{{{{{{{\bf{r}}}}}}}}}{{r}^{5}}$$

is the host monopole–dopant quadrupole interaction, and

4 $${V}_{{{{{{{{\rm{mQ}}}}}}}}}^{{{{{{{{\rm{dh}}}}}}}}}=-\frac{e}{8\pi {\epsilon }_{0}{\epsilon }_{{{{{{{{\rm{r}}}}}}}}}}\frac{{{{{{{{\bf{r}}}}}}}}{{{{{{{{\bf{Q}}}}}}}}}^{{{{{{{{\rm{h}}}}}}}}}{{{{{{{\bf{r}}}}}}}}}{{r}^{5}}$$

is the host quadrupole–dopant monopole interaction. *ϵ* = *ϵ*_0_*ϵ*_*r*_ and *e* are dielectric permittivities and elementary charge, respectively, and **Q**^d/h^ is the quadrupole tensor of the dopant/host.

The last two components (quadrupole–monopole interaction) depend on the relative orientation of a given dopant and host. Figure 2b shows *V*_C_ (multipoles–multipoles) along three representative relative orientations of hosts and dopants: along *x*, *y*, and *z* axes, where the *x*-axis is the direction where the molecule has the largest length (long axis), *z*-axis is the axis normal to the planar F_6_TCNNQ molecule (normal axis), and the *y*-axis is perpendicular to both (short axis), as depicted in Supplementary Fig. 1. In these cases the multipole-monopole interaction of Eq. (1) can be simplified to

5 $${V}_{{{{{{{{\rm{mQ}}}}}}}}}^{{{{{{{{\rm{dh}}}}}}}}}(\alpha \alpha )+{V}_{{{{{{{{\rm{mQ}}}}}}}}}^{{{{{{{{\rm{hd}}}}}}}}}(\alpha \alpha )=\frac{e}{8\pi {\epsilon }_{0}{\epsilon }_{{{{{{{{\rm{r}}}}}}}}}}\frac{1}{{r}^{3}}\left(-{Q}_{\alpha \alpha }^{{{{{{{{\rm{h}}}}}}}}}+{Q}_{\alpha \alpha }^{{{{{{{{\rm{d}}}}}}}}}\right)$$

with *α* = *x*, *y*, *z*.

Equation (5) shows that *V*_C_ has a linear dependence on the difference between quadrupole moments of the host and dopant. Depending on their sign, the quadrupoles of the host and dopant may therefore induce overscreening or underscreening. With the quadrupole moments of the host and dopant explored here (provided in Supplementary Table 3), overscreening $({V}_{{{{{{{{\rm{mQ}}}}}}}}}^{{{{{{{{\rm{dh}}}}}}}}}+{V}_{{{{{{{{\rm{mQ}}}}}}}}}^{{{{{{{{\rm{hd}}}}}}}}} > 0)$ therefore occurs for relative orientation along the normal axis (*z*-direction), and to a smaller extent along the short axis (*y* direction), as depicted in Fig. 2b. Relative orientation along the long (*x*) axis, in contrast, leads to the opposite effect $({V}_{{{{{{{{\rm{mQ}}}}}}}}}^{{{{{{{{\rm{dh}}}}}}}}}+{V}_{{{{{{{{\rm{mQ}}}}}}}}}^{{{{{{{{\rm{hd}}}}}}}}} < 0)$.

Notably, the microscopic model follows the trend of *z* and *y* alignment. This can be explained considering the anisotropy of both host and dopant: The minimal center-of-geometry distance of 3.5 Å is realized for relative orientation along the *z*-axis, while the closest distance for the underscreening case (relative orientation along the *x*-axis) is around 10 Å. The distribution of the orientation of host molecules relative to the long, short, and normal axis of the neighboring dopant molecules is shown in Supplementary Fig. 2. At the smallest distances, dopants and hosts tend to align (as stacks), e.g., are oriented along *z*-axes. While increasing host-dopant separation, there is a gradual transition towards distances where all relative orientations are equally probable. As a result, the *V*_C_ distribution at the small host–dopant distances is biased toward ${V}_{{{{{{{{\rm{mQ}}}}}}}}}^{{{{{{{{\rm{dh}}}}}}}}/{{{{{{{\rm{hd}}}}}}}}}(zz)$ and ${V}_{{{{{{{{\rm{mQ}}}}}}}}}^{{{{{{{{\rm{dh}}}}}}}}/{{{{{{{\rm{hd}}}}}}}}}(yy)$. This causes a reduction of ∣*V*_C_∣, i. e. a short-range overscreening effect.

To assess the relevance of this overscreening effect, we plot the distribution of distances from dopants to their nearest host (top of Fig. 2b). We see that 90% of the dopants have at least one host in their environment at a distance of *r* < 6.7 Å, where the overscreening effect is significant. In other words, 90% of the ICTC, which would be considered a deep trap using classical Coulomb interaction, have a relatively shallow ICTC binding energy.

### Tuning the overscreening effect to yield efficient dopants

**Fig. 3:**
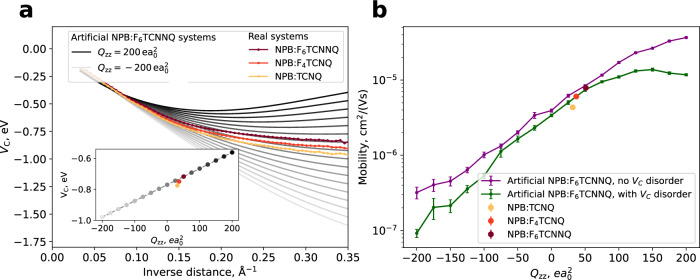
Tuning hole mobility using short-range overscreening effect. **a** Distance-dependent *V*_C_ for NPB doped with 5 mol% TCNQ, F_4_TCNQ, F_6_TCNNQ (color) and for dopants with artificially modified quadrupole *Q*_*zz*_ between −200 $e{a}_{0}^{2}$ and 200 $e{a}_{0}^{2}$ (gray, darker means larger *Q*_*zz*_ in steps of $25\,e{a}_{0}^{2}$). An increase in overscreening is observed for positive quadrupoles, negative quadrupoles diminish the overscreening. At a host–dopant distance of 5.3 Å (distance with the highest probability, see the top of Fig. 2b), *V*_C_ depends linearly on *Q*_*zz*_ (inset) and shows a variation of 0.4 eV. **b** Mean and its standard error of the hole mobility (points and error bars, respectively) in dependence of *Q*_*zz*_ shows a variation over two orders of magnitude, indicating an important dimension in the design of dopants.

To illustrate how to use the overscreening effect to enhance doping efficiency, we investigate NPB doped with 5 mol% of either TCNQ, F_4_TCNQ, or F_6_TCNNQ. The molecular structures are depicted in Fig. 1a, b. Figure 1c depicts the distribution of F_6_TCNNQ molecules in the NPB matrix. For these materials, *Q*_*zz*_ component of the quadrupole tensor is $31.56,\,36.79,\,\,{{\mbox{and}}}\,\,50.98\,e{a}_{0}^{2}$ for TCNQ, F_4_TCNQ, and F_6_TCNNQ, respectively (see Supplementary Table 3), i.e., *Q*_*zz*_ increases with the number of F-atoms. For these three systems, we computed the ICTC binding energy for over 1 million pairs in various morphologies at different distances. The distance-dependent averages of the resulting distributions are depicted in Fig. 3a (colored lines). We observe an increase of the overscreening effect with increasing *Q*_*zz*_, i.e., the larger the *Q*_*zz*_, the smaller the ∣*V*_C_∣ in the short-range.

For a systematic study of the dependence of *V*_C_ on dopant quadrupoles, we artificially modified the dopant’s quadrupole moment in the system NPB:F_6_TCNNQ from −200 to 200 $e{a}_{0}^{2}$. To ensure that the quadrupole tensor remains traceless (to preserve the fundamental electrostatics laws), *Q*_*xx*_ and *Q*_*yy*_ are changed proportionally to *Q*_*zz*_ (see also Supplementary Fig. 7). The resulting distance-dependent means of *V*_C_ are fitted to a third-order polynomial (to smooth out small features stemming from the NPB:F_6_TCNNQ morphology) and depicted in Fig. 3a in addition to the real systems. The unfitted means of the artificial systems are displayed in Supplementary Fig. 3. We find that a modification of the quadrupole tensor can tune the shape of *V*_C_(*r*). Notably, even small values of ${Q}_{zz}=-200\,e{a}_{0}^{2}$ do not diminish the effect of the short-range overscreening completely due to the host molecule’s quadrupole moment (see Eq. (1)), which we did not modify.

The inset of Fig. 3a shows *V*_C_ in dependence of *Q*_*zz*_ at a distance of 5.9 Å, which is the most probable distance from the dopant to the first neighboring host molecule. We find that *V*_C_ at this distance can be tuned by 0.4 eV by varying *Q*_*zz*_.

To quantify the impact of the dopant quadrupole tensor on doping efficiency, we computed the charge carrier mobility for doped NPB layers, including both real dopants and the ones with artificially modified *Q*_*zz*_. The mobility is computed using a kinetic Monte Carlo (kMC) protocol^32^ based on material-specific parameters (ICTC binding energy distributions, transport energy levels (IP), site distribution, etc.) derived from the multiscale workflow. Details are given in the “Method” section “Kinetic Monte Carlo method”. Figure 3b depicts the hole mobility in dependence of *Q*_*zz*_. The observed steady increase of the mobility with increasing *Q*_*zz*_ indicates that modification of *Q*_*zz*_ in the applied range allows controlling mobility by two orders of magnitude.

For the systems with artificially modified quadrupole, we further computed the mobility (I), taking into account the distribution of the ICTC binding energies *V*_C_ at every given *r* modeled as a Gaussian distribution (denoted as “with *V*_C_ disorder” in Fig. 3) and (ii) using only the mean values of *V*_C_(*r*) at a given distance (“no *V*_C_ disorder”). We find that applying a distance-dependent distribution of *V*_C_(*r*) rather than the distance-dependent mean leads to a reduction of the mobility especially for large *Q*_*zz*_: an increase of the quadrupole tensor components leads to a broadening of *V*_C_, and slightly hindering the effect of the short-range overscreening (see almost flat region between *Q*_*zz*_ = 75 and 200 $e{a}_{0}^{2}$, where the mobility does not grow albeit increasing *Q*_*zz*_).

### Experimental validation

**Fig. 4:**
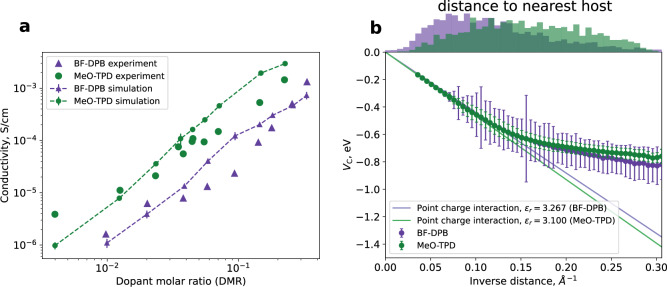
Experimental validation and the role of ICT state binding energy disorder. **a** Dependence of the simulated conductivities on the dopant molar ratio of the systems BF-DPB:F_6_TCNNQ and MeO-TPD:F_6_TCNNQ in comparison to experiment. Simulations reproduce experimentally observed absolute values and the concentration dependence of the conductivity. Points and error bars denote the mean and standard error of the mean of 20 independent kMC simulations. **b** Distance-dependent mean and disorder of *V*_C_(*r*) of the microscopic model (points and error bars, respectively) in comparison to a screened monopole–monopole model (classical Coulomb interaction) show (i) a much larger disorder of *V*_C_(*r*) for BF-DPB in comparison to MeO-TPD, explaining the reduced conductivity in (**a**) and (ii) a reversal of the order of *V*_C_(*r*) between the two host materials at small distances.

To validate our model of doped layers based on distributions of *V*_C_ we use a kMC protocol to compute the conductivity of BF-DPB:F_6_TCNNQ and MeO-TPD:F_6_TCNNQ (molecular structures depicted in Fig. 1a and b) in dependence of dopant concentrations, and compare the simulated results to experimental data^34^. As depicted in Fig. 4a we find good agreement of both absolute values and concentration dependence for both systems. Further, we resolve the experimentally observed difference between the material combinations, i.e., a larger conductivity of doped MeO-TPD in comparison to BF-DPB over the whole concentrations range. We note that for the computation of the conductivity shown in Fig. 4a, we have taken into account the concentration-dependence of IP_host_ and EA_dop_, which is highly sensitive to the dopant molar ratios (see “Methods” Section “Electronic properties of organic layers”).

The experimental observation of a higher conductivity in doped MeO-TPD compared to BF-DPB is counter-intuitive at first glance, as the intrinsic mobility of MeO-TPD (2.3 cm^2^/(Vs)) is lower than the mobility of BF-DPB (5.7 cm^2^/(Vs))^34^, both materials have approximately the same material density and the dopants have a high ionization ratio in both systems: In the low and medium doping regime (DMR < 10 %) the ratio is over 90% for MeO-TPD and over 75% for BF-DPB, see Supplementary Fig. 6.

An explanation of this effect is the different distance-dependent standard deviation of *V*_C_ (hereinafter, the disorder of *V*_C_) of both materials, depicted in Fig. 4b, along with mean values of *V*_C_(*r*), and comparison to *V*_C_(*r*) computed for classical point-charge interaction with the material specific dielectric permittivity computed for undoped systems^29^. We find that the disorder of *V*_C_ of BF-DPB exceeds that of MeO-TPD by over a factor of four, and is larger than the BF-DPB intrinsic disorder of the host HOMO. It is, therefore, a significant contribution to the total energy disorder^17^, and consequently reduces the conductivity of the doped BF-DPB below the conductivity of MeO-TPD.

Notably, for small distances, the order of *V*_C_(*r*) between the materials differs between the model based on microscopic distributions and the classical monopole-monopole interaction model. In the latter, the *V*_C_(*r*) differs only by the permittivity of the materials. This implies that the orientation and microelectrostatics of the host-dopant pairs have a larger impact on doping efficiency than the macroscopic screening. This is underlined by the comparison of the conductivity computed with *V*_C_(*r*) via screened monopole-monopole interaction^17,20,35^ with the conductivity from our model and experimental data (Supplementary Fig. 5). The conductivity computed with *V*_C_(*r*) by monopole–monopole interaction is very similar for the materials BF-DPB and MeO-TPD, hence the experimental observation of MeO-TPD:F_6_TCNNQ having a higher conductivity is not captured.

## Discussion

In this study, we showed that a relative orientation and microelectrostatics, in particular, quadrupole moments, of dopant and host molecules lead to a short-range overscreening effect, i.e., a decrease of the ICTC binding energy of activated host-dopant pairs at short distances. This effect implies that short host-dopant distances do not necessarily result in stronger interaction, in contrast to the intuitive expectation that an increase of host-doping spacing, e.g., by larger host molecules, decreases Coulomb interactions. The opposite may be the case: a large positive quadrupole moment of the dopant may even decrease ∣*V*_C_∣ at short distances. The short-range overscreening effect is equivalent to the dielectric constant going up at small host-dopant distances. In particular, the interaction between charged host and dopant molecules can be represented as an interaction between two point charges in a medium with increasing dielectric constant at smaller distances.

These findings, in line with the impact of the disorder of the ICTC binding energy, demonstrate that the material-specific microelectrostatics of host-dopant pairs is an essential ingredient to be included in computational models of OS such as ref. ^5,36^ to accurately reflect the effect of doping and its impact on device performance. Specifically, the width of distant-dependent *V*_C_ distribution (a disorder of *V*_C_), which up-to-date could only be experimentally measured and was shown to correlate with the activation energy^21^, is as important as the HOMO/LUMO energy disorder.

In general, microelectrostatics of OS molecules, in particular dipole and quadrupole moments, cause a range of effects, such as the reduction of the open-circuit voltage in OSC, that have paramount importance in the field of organic electronics^37–39^. In this work, we extend this list with a short-range overscreening effect and provide a computational tool to reliably quantify the ICTC binding energy, a key property for the design of efficient host–dopant material combinations.

We note that this study investigates ICT only^15,20^, although partial transfer may be possible for specific host–dopant pairs. Further, the model presented here was specifically designed to study effects in amorphous small molecule OS, e.g., by assuming that polarons are always localized on a single molecule, and delocalization of polarons is not considered. Investigation of effects in high-mobility or crystalline OS requires a different approach^40^.

In this work, we investigated the ICTC binding energy in prototypical host-dopant materials for OS applications. We found that at short distances, this binding energy is significantly reduced due to a short-range overscreening effect induced by quadrupole-monopole interactions.

This effect can be exploited in the design of efficient host-dopant combinations by pairing molecules with optimized microelectrostatics and relative orientation at small distances. In particular, we demonstrated that charge transport in doped layers could be tuned by up to two orders of magnitude by modification of the dopant quadrupole moment.

Further, we found that the disorder of the ICTC binding energy, i.e., the width of the distribution at a certain distance, has a strong impact on conductivity, up to the extent of typically considered HOMO/LUMO disorder. Specifically, we demonstrated that the ICTC binding energy disorder is strongly material-dependent and can even invert the order of two materials in terms of conductivity when doped. By comparing computed conductivity to experimental data, we showed that the ICTC binding energy disorder needs to be taken into account to accurately predict charge transport in doped OS.

The proposed multiscale model allows for determining both the distance-dependent mean and disorder of the ICTC binding energy, taking into account molecular orientation and polarization effects on a microscopic level. As the ICTC binding energy is a key quantity to assess the quality of host-dopant combinations, this approach is an efficient and reliable tool to systematically analyze doping performance and to enable rational design of materials for doped OS in silico.

## Methods

### Atomistic morphologies

Materials morphology is generated with the method DEPOSIT^41,42^, a Monte Carlo-based protocol that mimics physical vapor deposition. Single molecules (randomly choosing either host or dopant according to their concentration) are added to the simulation box (180 Å × 180 Å square) one at a time and use a Metropolis Monte Carlo-based basin hopping via simulated annealing (SA)^43^ to scan the morphology surface and relax into their thermodynamic equilibrium configuration. Specifically, we repeatedly annealed from 4000 K to 300 K in 76 SA cycles with 50,000 Monte Carlo steps each, where each step is either a rigid translation, rigid rotation, or the rotation of a single dihedral angle. After the equilibration of a molecule on the substrate, the next molecule is added to generate morphologies of 10,000 molecules per sample. Periodic boundary conditions in the *x*- and *y*-axis were applied to ensure also bulk properties of the morphology at the edge; z is the direction of morphology growth - no boundary conditions are applied to this axis. Depending on the size of the molecules, the size of the morphology, the *z*-length varies but is always larger than 240 Å. Optimal vacuum configurations of the molecules are generated with DFT (basis set def2-SV(P)^44^ and functional BP86^45^). During the deposition, the interaction between the deposited molecule and the substrate is modeled using Lennard–Jones potentials and Coulomb potential based on ESP charges. The intramolecular interaction (i.e., the energy of various configurations of flexible molecules NPB and TCTA) is modeled using molecule-specific force fields derived by step-wise rotation of single dihedral angles of the molecules in vacuum and computing the DFT energies of each configuration using def2-SV(P) basis set and BP86^45^ exchange-correlation functional. All DFT computations of this work are done using the DFT package Turbomole^46^ and the resolution-of-the-identity approximation^47,48^ if not noted otherwise.

Supplementary Table 2 includes the density of intrinsic host molecules morphologies (NPB, MeO-TPD, and BF-DPB).

All morphologies used in this work were deposited by this method: NPB is doped with TCNQ, F_4_TCNQ, F_6_TCNNQ: 5 different morphologies, with each having a 5% dopant molar ratio, were deposited.

The hole transport materials MeO-TPD and BF-DPB both are doped with F_6_TCNNQ in different dopant molar ratios. For each DMR, seen in Fig. 4a), two morphologies were created.

### Electronic properties of organic layers

The simulated morphologies need to be characterized in their electronic properties to proceed with charge-transport simulations. More specifically, the rate of an integer CT depends on the following quantities, which are part of the Marcus theory.

Computing IP and EA is done as follows: IP_host_ of host molecules defines the charge transport levels of holes. Hole CT between host molecules is not influenced by the absolute energetic level, but its disorder *σ*_IP,host_. However, the CT of electrons between the host and dopant is influenced by the difference between IP_host_ and EA_dop_, further labeled as Δ*E*_IP,EA_, which determines the rate and balance of dopant ionization. For the simulations in Section “Tuning the overscreening effect to yield efficient dopants” Δ*E*_IP,EA_ is set to 0.2 eV to ensure an almost 100% dopant ionization. For the systems BF-DPB:F_6_TCNNQ and MeO-TPD:F_6_TCNNQ of Section “Experimental validation” IP_host_ and EA_dop_ are computed according to our previous work^29^ for different DMR to account for concentration-dependent shifts of IP and EA levels reported previously^49^. The polarization energy *P*^(+/−)^(*R*) of 5 embedded core molecules is computed for radii *R* ∈ [20, 36] Å and extrapolated to infinity to obtain the bulk value. The shell of charge equilibration is 40 Å around the core molecules. For BF-DPB:F_6_TCNNQ the Δ*E*_IP,EA_ is computed for DMR values of 0.994%, 5.851%, and 33.093%, for MeO-TPD:F_6_TCNNQ at 0.400%, 4.474%, and 22.694%. Linear interpolation was applied to get Δ*E*_IP,EA_ for other DMR values, see Supplementary Table 4 and Supplementary Fig. 4. Notably, Δ*E*_IP,EA_ between the lowest and the highest DMR in the system BF-DPB:F_6_TCNNQ differs by about 200 meV.

The disorder of IP_host_ is determined by a fitting method as this work aims to simulate the interaction of host and dopant molecules in OS by ab initio methods, but not the intrinsic energy disorder parameter. This is done to exclude a possible source of inaccuracy, which is not directly related to the present work. Hence, for NPB, we used a literature value of the disorder^50^. For the other materials, we used the here described multiscale workflow, including kMC simulations of intrinsic OS to fit the disorder *σ*_IP,host_ to experimental mobility. The obtained disorder is then used in simulations of the doped OS of the respective host molecule.

For this fit intrinsic morphologies were simulated for different electric fields. The zero-field mobility is obtained by a linear fit of the log(mobility) versus the square root of the electric field, see Supplementary Figs. 8 and 9. These zero-field mobilities for different disorders are used to fit the disorder to the experimental mobility, see Supplementary Figs. 10 and 11. The obtained disorders are shown in Supplementary Table 2.

The transfer integrals/ electronic couplings are calculated for 300 pairs of molecules. For that, a self-consistent equilibration of the charge densities of the individual molecules in the morphology is simulated^31^ with def2-SVP and BP86 as basis set and functional, respectively. The electronic coupling of the hole transfer between two molecules is calculated with the approach of Stehr et al.^51^:

6 $${J}_{if}=\frac{{\hat{H}}_{if}-\frac{1}{2}\left({\hat{H}}_{ij}+{\hat{H}}_{jj}\right){S}_{ij}}{1-{S}_{ij}^{2}},$$

with

7 $${\hat{H}}_{ij}=\left\langle {\phi }_{i}\left|{\hat{H}}_{{{{{{{{\rm{KS}}}}}}}}}\right|{\phi }_{j}\right\rangle,{S}_{ij}=\left\langle {\phi }_{i}|{\phi }_{j}\right\rangle,$$

where *ϕ*_*i*_ and *ϕ*_*j*_ are the highest occupied molecular Kohn–Sham orbitals of the molecule *i* and *j* in an extended dimer basis, ${\hat{H}}_{{{{{{{{\rm{KS}}}}}}}}}$ the Kohn-Sham operator of the neutral molecular dimer, and *S*_*ij*_ the overlap of the orbitals. The transfer integrals in the kMC simulations are drawn from distance-dependent probability distributions matching the microscopic calculations.

The vacuum reorganization energy of the molecules is calculated with the Nelsen four-point procedure^52^; see Supplementary Table 2. The geometry optimization was done using def2-SVP^44^ and B3LYP^53–55^. The single point energy was computed using def2-TZVP^44^ and B3LYP. For NPB, also the reorganization energy of 7 embedded molecules (in intrinsic NPB morphologies)is computed as follows. Using Nelson’s four-point procedure, geometry relaxations for charged and neutral states are computed with an additional constraining potential caused by neighboring molecules in the morphology. The environment constraints are introduced by representing (fixed) atoms of neighboring molecules by effective core potentials^56^ of the respective period (e.g., neighboring carbon and nitrogen atoms are represented by the same ECP in this approach). The ratio between the embedded value and the vacuum value for NPB is found to be approximately 0.7. We, therefore, use a reorganization energy of 0.7 times the computed vacuum value for all host molecules. For all dopants, we set the reorganization energy to 0.155 eV.

The dielectric permittivity, *ϵ*_*r*_, is computed by the quantum embedding method QuantumPatch according to our previous work^29^. For computing *ϵ*_*r*_ the polarization energy of 5 embedded molecules is computed.

### Computing the Coulomb binding energy

The ICT state binding energy *V*_C_ is computed according to^57^:

8 $${V}_{{{{{{{{\rm{C}}}}}}}}}={E}_{{{{{{{{\rm{CT}}}}}}}}}-\left({{{{{{{{\rm{IP}}}}}}}}}_{{{{{{{{\rm{host}}}}}}}}}-{{{{{{{{\rm{EA}}}}}}}}}_{{{{{{{{\rm{dop}}}}}}}}}\right),$$

using the quantum embedding method QuantumPatch. Here, *E*_CT_ is the change in the system energy upon electron transfer from the host to the dopant (ICT state creation), also called embedded charge separation energy. A description of how to compute IP, and EA of embedded molecules by the QuantumPatch method is found in ref. ^29^. This method can be transferred to compute *E*_CT_: the space is divided into two parts (shells): (1) the explicit shell in which explicit quantum embedding calculations take place, and (2) the implicit shell treated according to classical electrostatics. Explicit space is thereby the union of two spheres around the host–dopant molecule pair (Fig. 1f). The space outside the union is an implicit shell, see Supplementary Fig. 15. One time the electron density equilibration of the molecules is done for all molecules in the neutral state and one time for the host–dopant pair in the ICT state (host is positively charged, dopant is negatively charged). The explicit part of the ICT state energy ${E}_{{{{{{{{\rm{CT}}}}}}}}}^{{{{{{{{\rm{expl}}}}}}}}}$ is given by the energy difference between those two systems’ energies:

9 $${E}_{{{{{{{{\rm{CT}}}}}}}}}^{{{{{{{{\rm{expl}}}}}}}}}={E}_{f}^{{{{{{{{\rm{expl}}}}}}}}}-{E}_{i}^{{{{{{{{\rm{expl}}}}}}}}},$$

where ${E}_{i}^{{{{{{{{\rm{expl}}}}}}}}}$ is the energy of the uncharged (initial) and ${E}_{f}^{{{{{{{{\rm{expl}}}}}}}}}$ of the CT (final) state. The implicit part of the energy ${E}_{{{{{{{{\rm{CT}}}}}}}}}^{{{{{{{{\rm{impl}}}}}}}}}$ is derived using classical electrostatics (ICTC is approximated as a dipole):

10 $${E}_{{{{{{{{\rm{CT}}}}}}}}}^{{{{{{{{\rm{impl}}}}}}}}}=\frac{1}{2{\epsilon }_{0}}\left(1-\frac{1}{{\epsilon }_{r}}\right){\int}_{V}{\left|{\overrightarrow{D}}^{{{{{{{{\rm{dip}}}}}}}}}(\overrightarrow{r})\right|}^{2}{{{{{{{\rm{d}}}}}}}}V,$$

where *V* is the volume of the implicit space having a relative dielectric permittivity of *ϵ*_*r*_ and ${\overrightarrow{D}}^{{{{{{{{\rm{dip}}}}}}}}}$ denotes the electric displacement field produced by the ICTC. This integral is solved numerically. *ϵ*_*r*_ is computed beforehand by the quantum embedding method QuantumPatch according to^29^. The final ICT state energy is thus given by

11 $${E}_{{{{{{{{\rm{CT}}}}}}}}}={E}_{{{{{{{{\rm{CT}}}}}}}}}^{{{{{{{{\rm{expl}}}}}}}}}+{E}_{{{{{{{{\rm{CT}}}}}}}}}^{{{{{{{{\rm{impl}}}}}}}}}.$$

*V*_C_ is assumed to be much more accurate than the accuracy of single-point DFT calculations as the DFT errors cancels out by computing *V*_C_ (Eq. (8)).

The basis set and functional used for individual single point computations are def2-SVPD^58^ and BP86^45^. The explicit polarization shell has a radius of 30 Å.

### Estimation of the Coulomb binding energy *V*_C_ distribution

The quantum embedding method QuantumPatch is accurate but computationally extensive. We applied it to compute *V*_C_ for 30 host–dopant pairs for this reason. A *V*_C_ distribution of a statistically relevant number of host-dopant pairs that would be sampled by possible host-dopant orientations, distances, and conformations can be employed using simplified methods. We have employed a range of methods that, despite evaluating host–dopant Coulomb interactions at a simplified level, contain parameters of stand-alone host and dopant molecules, which can be efficiently computed. Every such method has three components to represent the Coulomb interactions in an integer CTC: electrostatics of the host (1), electrostatics of the dopant (2), and effect of the polarizable environment, e.g., organic matrix (3). Surprisingly, accurate estimation is obtained as follows. The electrostatics of the dopant anion is represented as a sum of the first three moments of the multipole expansion of its electron density: monopole, dipole, and quadrupole moments (the dipole moment of all TCNQ explored derivatives are negligible). The host cation is represented as ESP partial charges^59^. Finally, the effect of the polarizable environment is reflected by describing multipole–partial charges interaction in the effective continuous medium with relative permittivity *ϵ*_*r*_. The latter is computed for actual morphologies according to ref. ^29^.

The formula to compute this estimation reads:

12 $${V}_{{{{{{{{\rm{C}}}}}}}}}({{{{{{{\rm{estimated}}}}}}}})=\mathop{\sum}\limits_{i}-\frac{e{q}_{i}^{{{{{{{{\rm{h}}}}}}}}}}{4\pi {\epsilon }_{0}{\epsilon }_{{{{{{{{\rm{r}}}}}}}}}{r}_{i}}+\frac{{q}_{i}^{{{{{{{{\rm{h}}}}}}}}}}{8\pi {\epsilon }_{0}{\epsilon }_{{{{{{{{\rm{r}}}}}}}}}}\frac{{{{{{{{{\bf{r}}}}}}}}}_{i}{{{{{{{{\bf{Q}}}}}}}}}^{{{{{{{{\rm{d}}}}}}}}}{{{{{{{{\bf{r}}}}}}}}}_{i}}{{r}_{i}^{5}}$$

Here, *ϵ* = *ϵ*_0_*ϵ*_*r*_ and *e* are dielectric permittivity and elementary charge, respectively; *i* indexes partial charges of the host molecule; ${q}_{i}^{{{{{{{{\rm{h}}}}}}}}}$ is an *i*th partial charge of the host; **r**_*i*_ is a vector connecting host partial charges and dopant center-of-geometries; **Q**^*d*^ is a quadrupole tensor of the dopant.

This approximation to *V*_C_ is referred to as *V*_C_(estimated) in the main text and depicted in Fig. 2a, b.

Another method, described in the main text, represents the electrostatics of both the host and the dopant as multipole expansion up to a quadrupole moment. It is less accurate but more intuitive and generic. This approximation to *V*_C_ is used in the main text to plot three auxiliary lines in Fig. 2b.

### Kinetic Monte Carlo method

We model charge transport and doping activation using a kinetic Monte Carlo algorithm, where electrons and holes can hop between molecules. Hole transfers between HOMOs of molecules constitute hole transport, electron transfers from LUMOs of molecules account for electron transport, and electron transfers from host-HOMOs to dopant-LUMOs constitute dopant activation. We note that electron transfer from LUMO to LUMO is not considered in the simulations of Section “Tuning the overscreening effect to yield efficient dopants” as this is only relevant at high doping ratios. The organic material is described on molecular resolution, where each molecule is represented by its center-of-geometry, as calculated from the morphology generated by DEPOSIT. The state of the system (i.e., the position of charges) is propagated in time according to the rates of the individual integer CT processes. CT rates between molecules are calculated as:

13 $${\omega }_{AB}=\frac{\pi }{\sqrt{{\hslash }^{2}{k}_{B}T\lambda_{AB} }}{J}_{AB}^{2}\exp \left(-\frac{{\left(0.5\left(\Delta {E}_{AB}+{\lambda }_{AB}+|\Delta {E}_{AB}+{\lambda }_{AB}|\right)\right)}^{2}}{4{\lambda }_{AB}{k}_{B}T}\right)$$

where reorganization energies *λ*_*AB*_ and transfer integrals *J*_*AB*_ are used analogously to prior work^60^. Δ*E*, the energy difference between the final and initial state, is a consequence of molecule-specific IPs and EAs and the difference of the Coulomb energy of the final and initial charge configuration, i.e., the Coulomb interaction between all electrons and holes in the system for the given configuration. Long distance Coulomb interaction between charge carriers is calculated from the interaction of point charges, short distance Coulomb energy (nearest 250 neighbors) is drawn from tabulated Coulomb energy distributions, calculated for the specific system as described in Section “Estimation of the Coulomb binding energy *V*_C_ distribution”. Simulations to calculate electrical mobility/conductivity are done at 300 K and an applied field of 0.015 V nm^−1^. As stated in “Atomistic morphologies”, several (five in Section “Tuning the overscreening effect to yield efficient dopants”, and two in “ Experimental validation”) morphologies were produced for one set of material/simulation parameters. On each of these morphologies, we performed 20 independent kMC simulations. The results of mobility and conductivity that we present are averages over these simulations and morphologies. The respective Fig. 3b and 4a show the mean and standard error of the mean, correspondingly.

## Supplementary information

Supplementary Information

## Data Availability

The authors declare that all data supporting the findings of this study are available within the paper and its Supplementary Information files or available from the corresponding author upon request.

## Code availability

Upon a reasonable request, the authors will provide an academic single-user trial license of Deposit, LightForge, and QuantumPatch for the purpose of reproducing the results of this paper.
