# Supplementary material for: Controlling doping efficiency in organic semiconductors by tuning short-range overscreening
Source: Nat Commun. 2023 Mar 13;14:1356. doi: 10.1038/s41467-023-36748-x (PMC10008838; doi:10.1038/s41467-023-36748-x)
Supplement: Supplementary file 1 — Supplementary Information [file 41467_2023_36748_MOESM1_ESM.pdf]

Supplementary Information

Controlling doping efficiency in organic semiconductors by tuning short-range overscreening

Armleder et al.

Supplementary Figures 3
Supplementary Figure 1 3
Supplementary Figure 2 4
Supplementary Figure 3 5
Supplementary Figure 4 6
Supplementary Figure 5 7
Supplementary Figure 6 8
Supplementary Figure 7 9
Supplementary Figure 8 10
Supplementary Figure 9 10
Supplementary Figure 10 11
Supplementary Figure 11 11
Supplementary Figure 12 12
Supplementary Figure 13 13
Supplementary Figure 14 13

Supplementary Tables 14
Supplementary Table 1 14
Supplementary Table 2 15
Supplementary Table 3 16
Supplementary Table 4 16

Supplementary References 17

Supplementary Figures

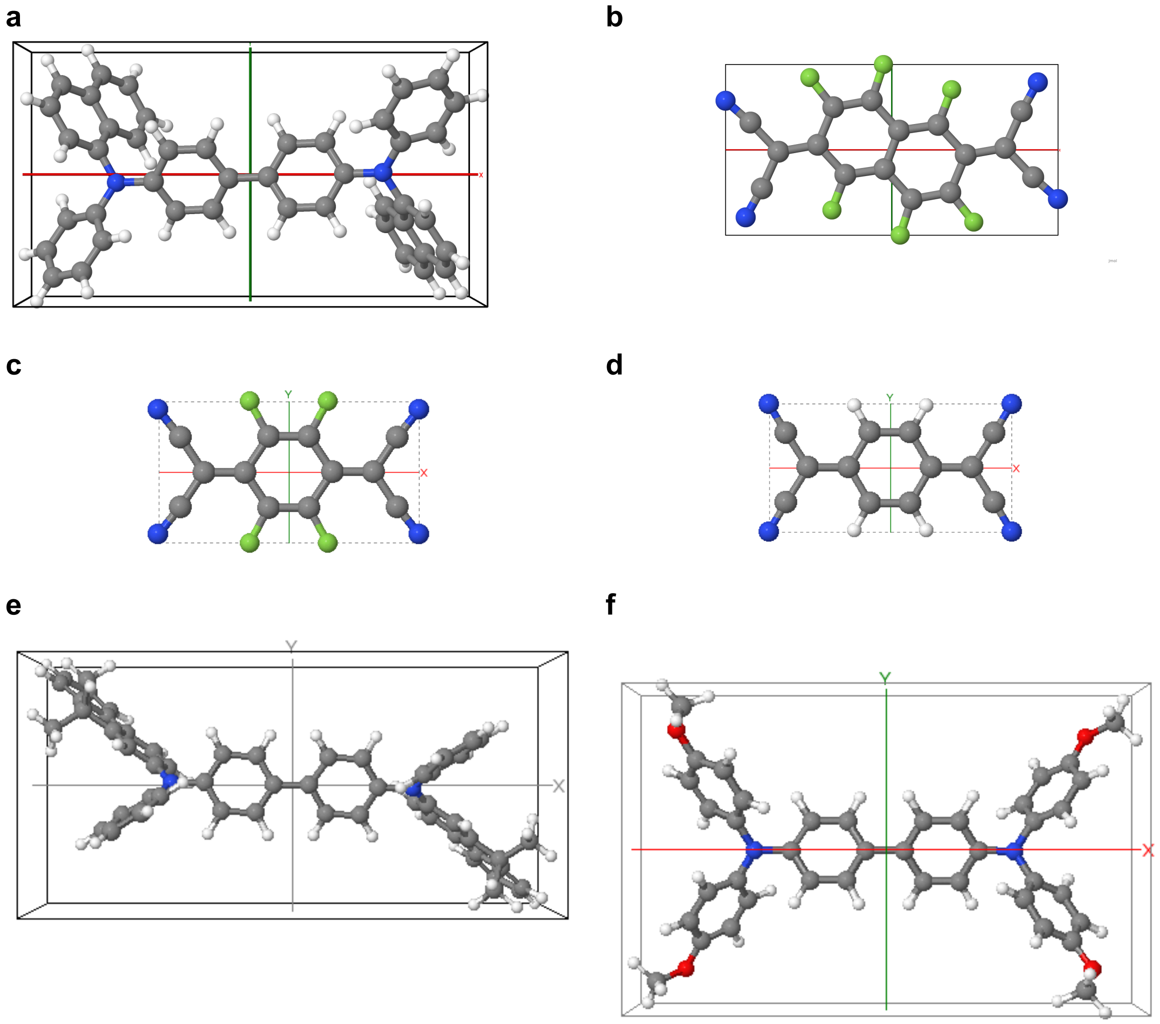

Supplementary Figure 1. Orientation of the host and dopant molecules relative to the coordinate system: (a) NPB, (b) F_6TCNNQ, (c) F_4TCNQ, (d) TCNQ, (e) BF-DPB, (f) MeO-TPD.

Axes orientation:
x axis: horizontal (red), it is called a “long axis”,
y axis: vertical (green), it is called a “short axis”,
z axis: perpendicular to the screen. It is called a “normal” axis.

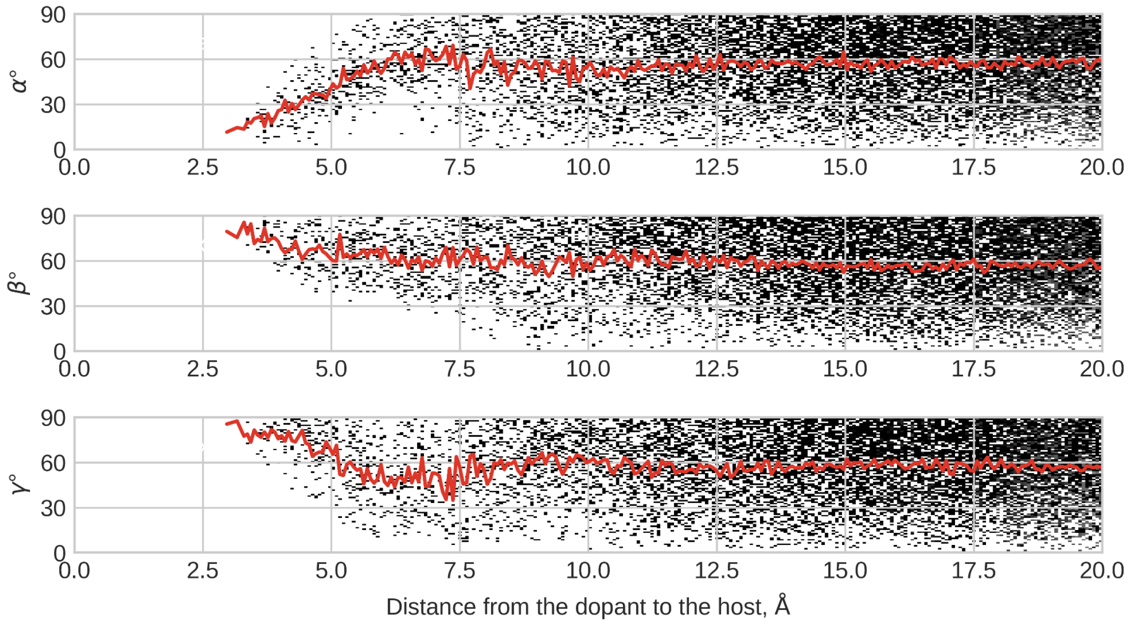

Supplementary Figure 2. Distribution of the angles between normal (α), short (β) and long (γ) axes of F_6TCNNQ molecule (see Supplementary Figure 1) and the vector connecting the dopant center of geometry (cog) and cog of the host molecule (NPB). For the reference: almost zero value of α, the normal-axis angle, means that the center of geometry of a given host is directly on top of the molecular plane of F_6TCNNQ, i.e. the two align perpendicular to the z-axis (dopant coordinate system). See Supplementary Figure 1 for details on molecule orientation relative to axes.

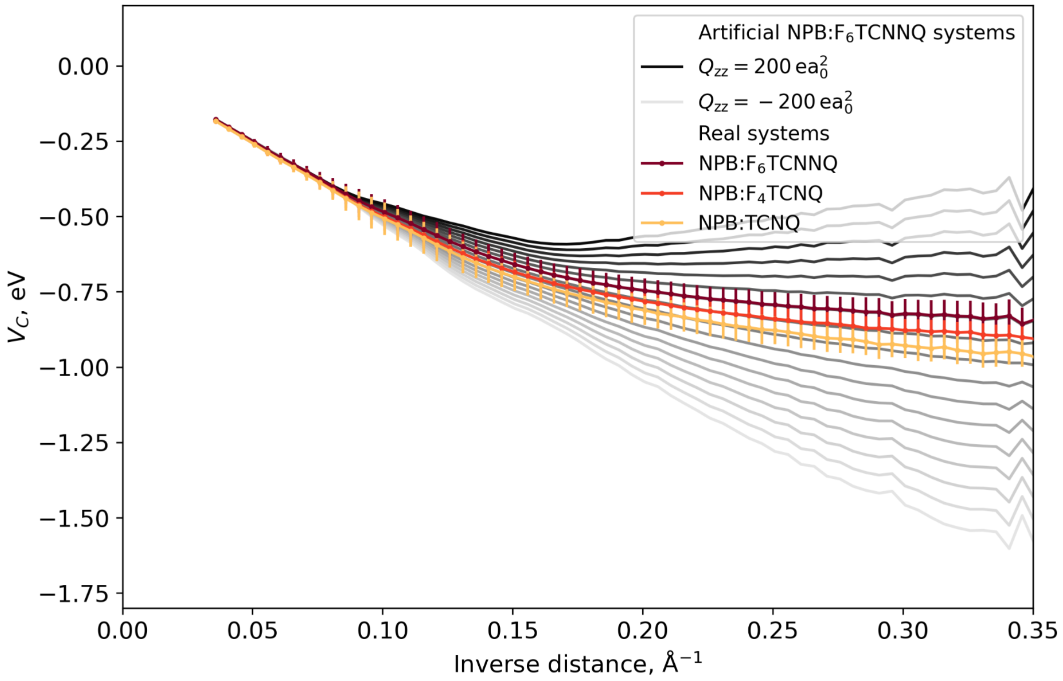

Supplementary Figure 3. Mean values of the distance dependent V_C for a NPB:F_6TCNNQ system with an artificially tuned dopant quadrupole moment and realistic systems (NPB doped with TCNQ, F_4TCNQ and F_6TCNNQ). The realistic systems are plotted with the V_C disorder (distance dependent standard deviation of the V_C (r) distribution, r being the host-dopant distance) as error bars.

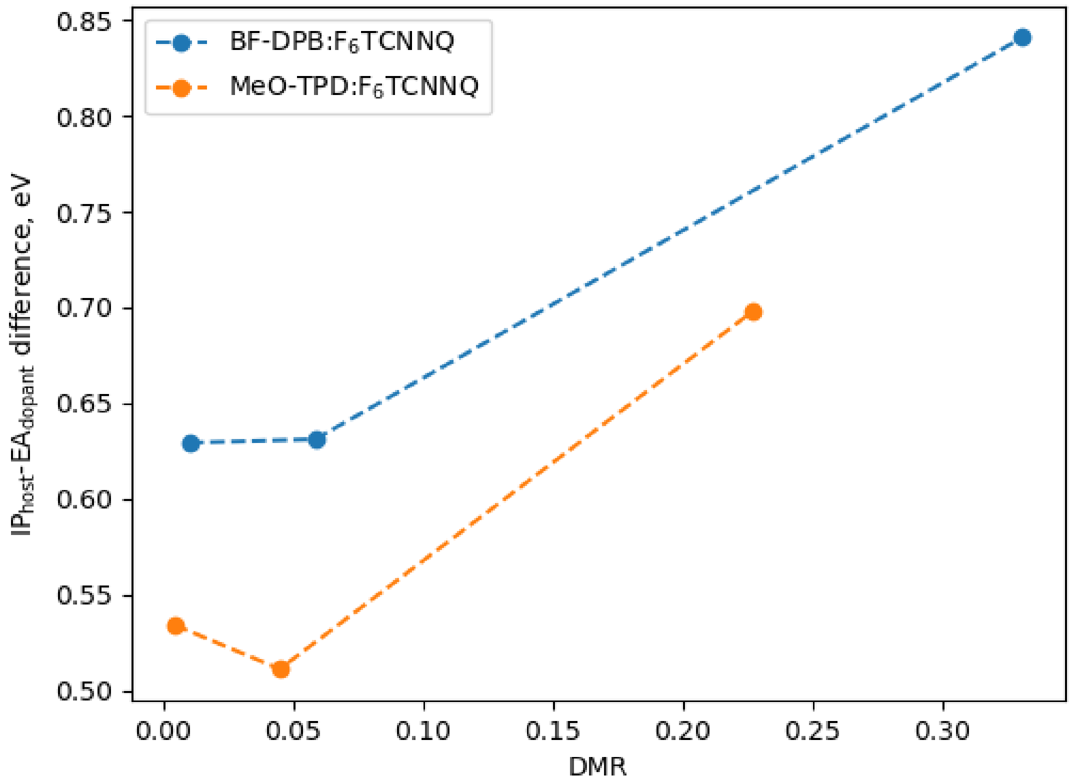

Supplementary Figure 4. $IP_{host} - EA_{dopant}$ as computed in Supplementary Table 4. We apply linear interpolation, indicated by the dashed lines to derive the input for kMC simulations in Section 2.3 for DMR values not explicitly computed.

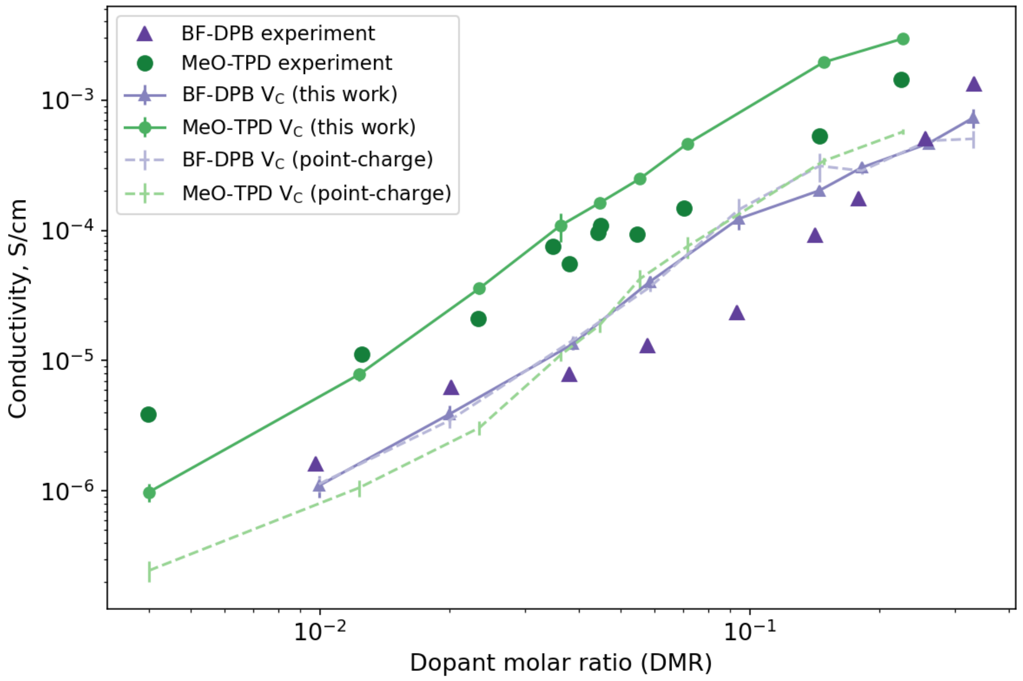

Supplementary Figure 5. Computed conductivity for BF-DPB:F_6TCNNQ and MeO-TPD:F_6TCNNQ in comparison with experimental data. "This work" depicts data where Coulomb binding energy was computed with the method introduced in the manuscript (host partial-charges <-> dopant multipole interaction). "point-charge" is data where V_C is the binding energy of two point charges with a distance according to the molecular center of geometries. The error bars denote the standard error of the mean. Other settings are described in the manuscript method Section 4. The computed conductivity using the point-charge model shows a negligible difference between the systems, in contradiction with experimental data. In contrast, simulations using the V_C of this work yield conductivities in good agreement with experimental data, most importantly in the correct order [3].

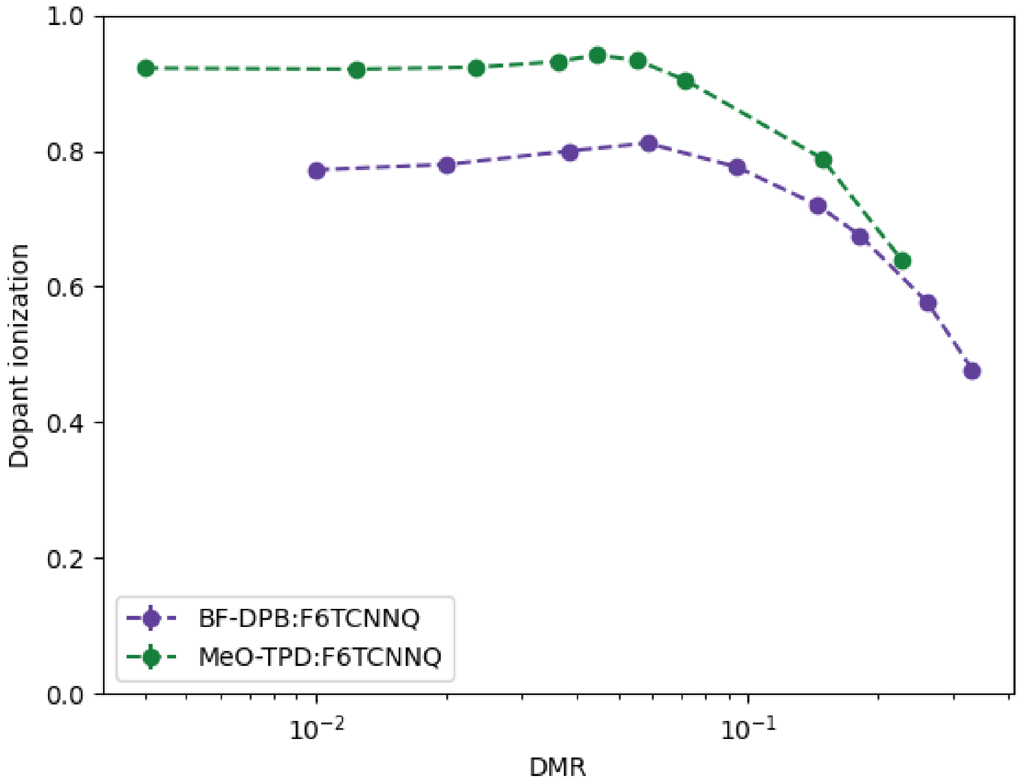

Supplementary Figure 6. Dopant ionization ratio in the systems BF-DPB:F_6TCNNQ and MeO-TPD:F_6TCNNQ in dependence of the dopant molar ratio, DMR. The conductivity in these systems vs. DMR are depicted at Figure 4a. The higher ionization ratio of MeO-TPD fits to experimental observation of MeO-TPD having a smaller IP than BF-DPB, 5.07 eV [3] and 5.23 eV [3], respectively.

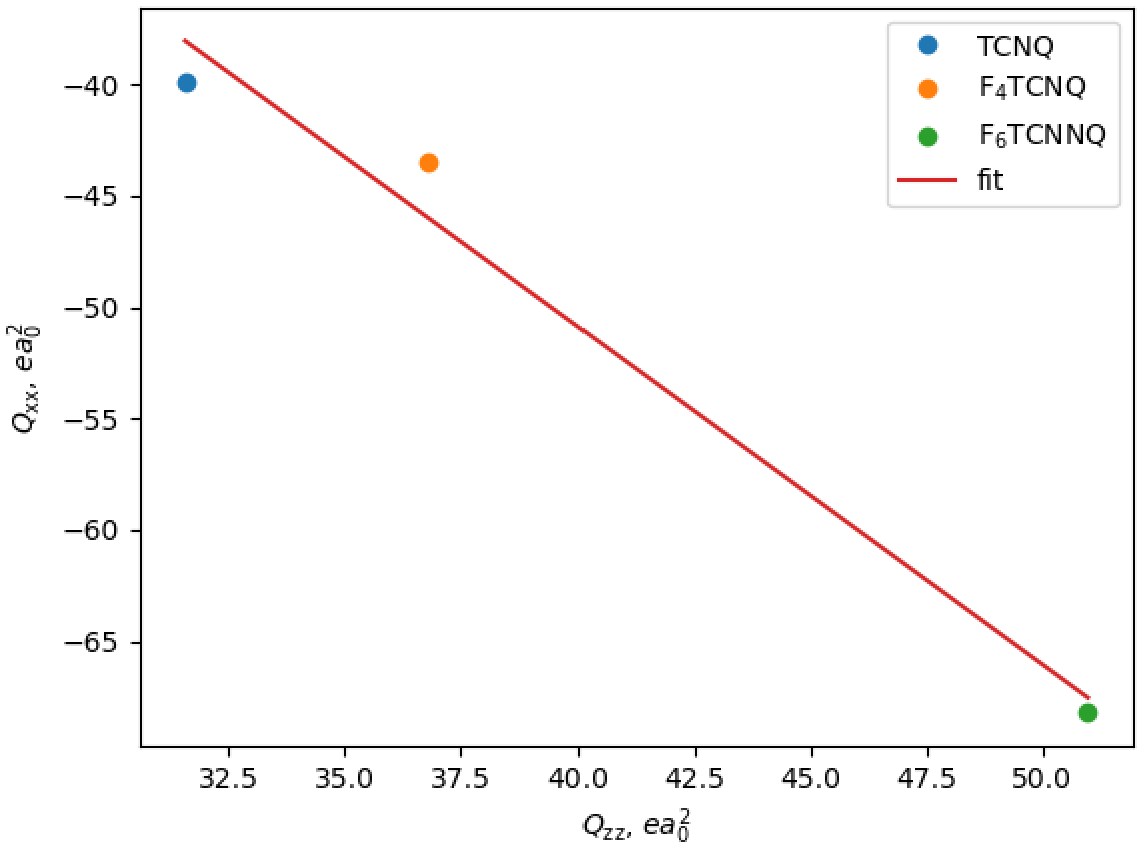

Supplementary Figure 7. zz-component of the quadrupole moment (Q) of the dopants vs. xx-components of the dopant quadrupole moment. The trend suggests a linear dependence between xx and zz-components. We used this dependency to construct “realistic” artificial dopants in Section 2.2, i.e. kept the ratio of xx and zz components constant when increasing the quadrupole moment.

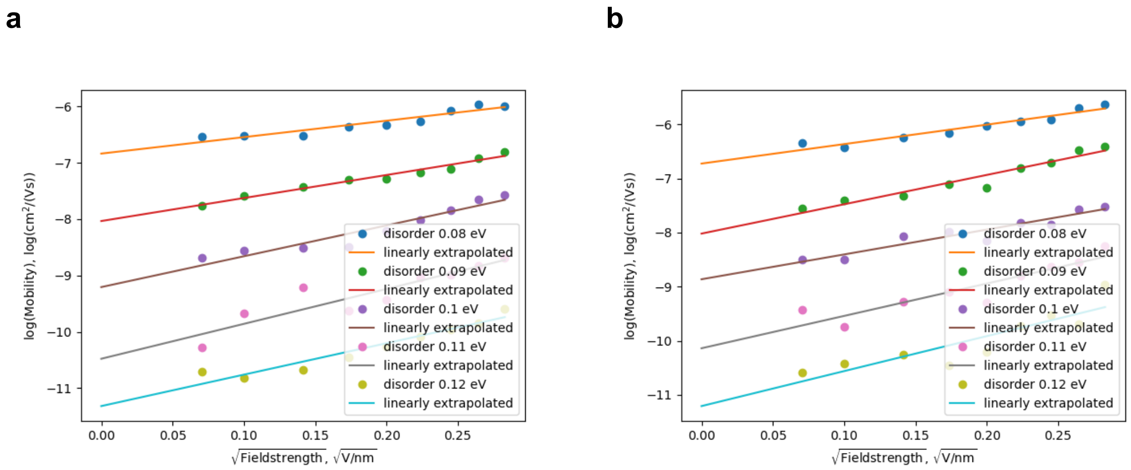

Supplementary Figure 8. Determining zero-field mobilities from intrinsic organic simulations for BF-DPB for two morphologies (**a** and **b**). The disorder denotes the standard deviation of the Gaussian distributed hole transport energy levels, i.e. the ionization potentials of BF-DPB. Simulation parameters are set according to the manuscript method Section 4. The applied field in the simulations is 0.005 V/nm.

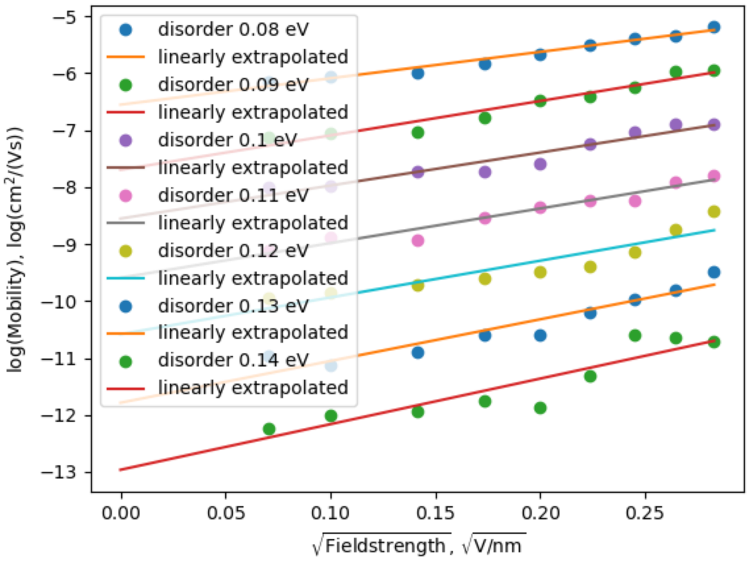

Supplementary Figure 9. Determining zero-field mobilities from intrinsic organic simulations for MeO-TPD. The disorder denotes the standard deviation of the Gaussian distributed hole transport energy levels, i.e. the ionization potentials of MeO-TPD. Simulation parameters are set according to the manuscript method Section 4. The applied field in the simulations is 0.005 V/nm.

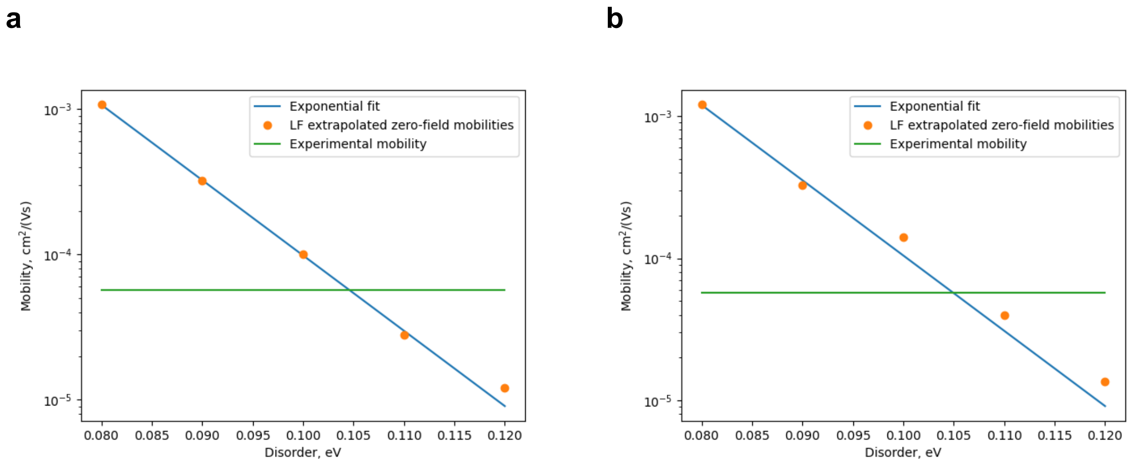

Supplementary Figure 10. Fit of the intrinsic disorder to zero-field mobility, BF-DPB, for two generated morphologies, **a** and **b**. For **a**: extracted disorder = 0.1046 eV, for **b**: 0.1050 eV. Experimental mobility (target) is $5.7x10^{-5}$ $cm^2/(Vs)$ [3]. This shows the deviation between the different morphologies is small. Simulation parameters are set according to the manuscript method Section 4. The applied field in the simulations is 0.005 V/nm.

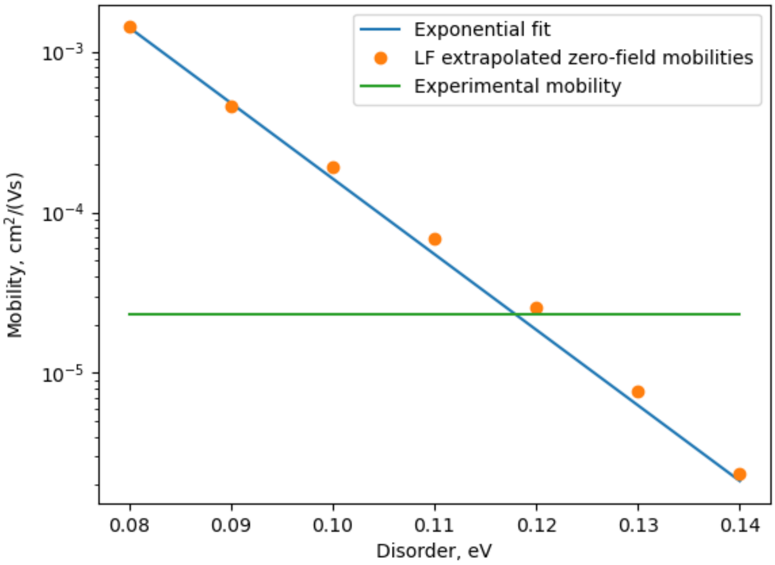

Supplementary Figure 11. Fit of the intrinsic disorder to zero-field mobility, MeO-TPD. Extracted disorder=0.1180 eV. Experimental mobility (target) is $2.3x10^{-5}$ $cm^2/(Vs)$ [3]. Simulation parameters are set according to the manuscript method Section 4. The applied field in the simulations is 0.005 V/nm.

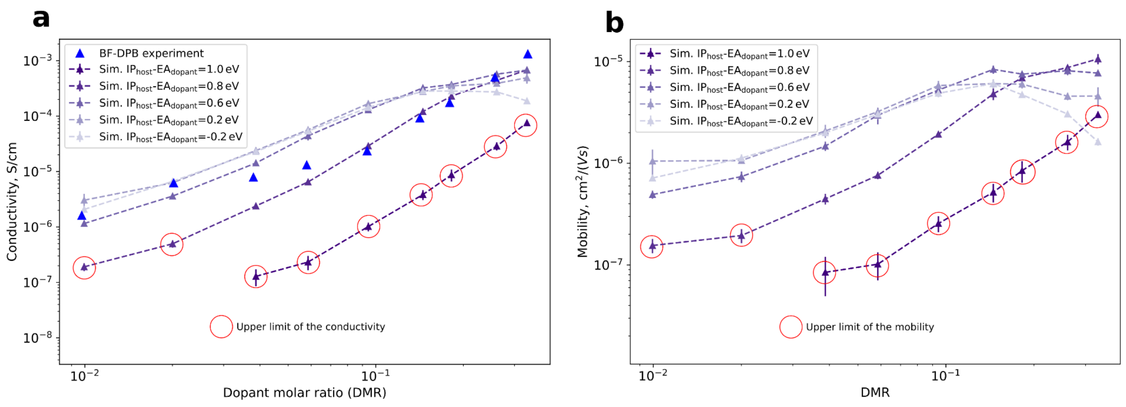

Supplementary Figure 12. Conductivity (a) and mobility (b) versus the dopant molar ratio for the system BF-DPB:F_6TCNNQ for different $IP_{host} - EA_{dopant}$ differences. Simulation parameters are set according to the manuscript method Section 4. A large difference (e.g. of 1.0 eV) corresponds to “weak” dopants, when only a small fraction of dopants are ionized (see Supplementary Figure 13), fewer holes are generated and hence lower conductivity is expected. Decreasing the difference, increases the conductivity/mobility in the low doping regime as expected. For high dopant molar ratios, the conductivity/mobility may, however, decrease even though more charge carriers are generated. This happens for the “border-line” dopants, where $IP_{host} - EA_{dopant} \sim V_C$ (V_C is around 0.6 … 0.8 eV for both systems at relevant host-dopant distances). This is reminiscent of the condition where a CT salt becomes conductive [5], but in our case the conductivity increases marginally. At larger dopant ionization ratio the mobility drops as the number of cites available for hopping decreases (these are either occupied by a hole or an electron). This causes an overall saturation or drop in the conductivity for high DMR at low $IP_{host} - EA_{dopant}$ differences (i.e. for “strong” dopants). Points and error bars denote mean and standard error of the mean over all converged replica. We note that simulations with only partly converged replicas (3 days convergence time limit) are marked by a red circle. These points describe an upper limit of the conductivity as replicas with higher mobility converge faster. The partly converged simulations are especially found for large $IP_{host} - EA_{dopant}$ differences as the mobility is generally small there (b). For unmarked points 80 out of 80 replicas have converged (two morphologies, each having 40 replica).

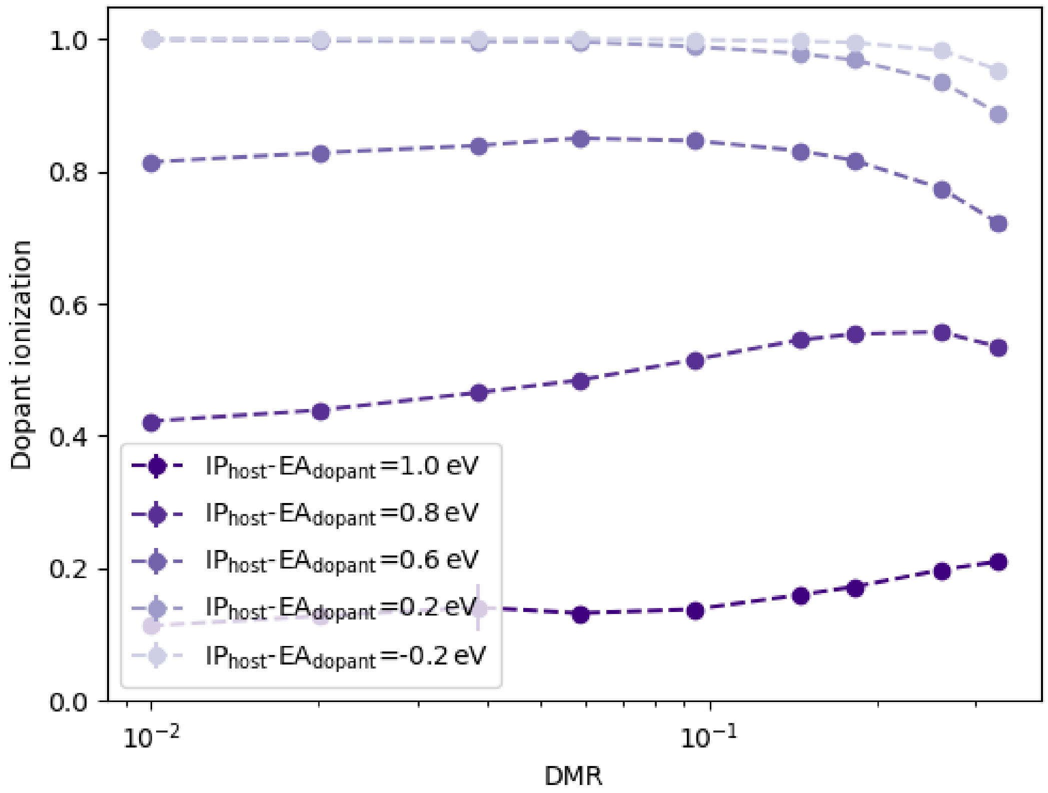

Supplementary Figure 13. Dopant ionization corresponding to the kMC simulations shown at Supplementary Figure 12.

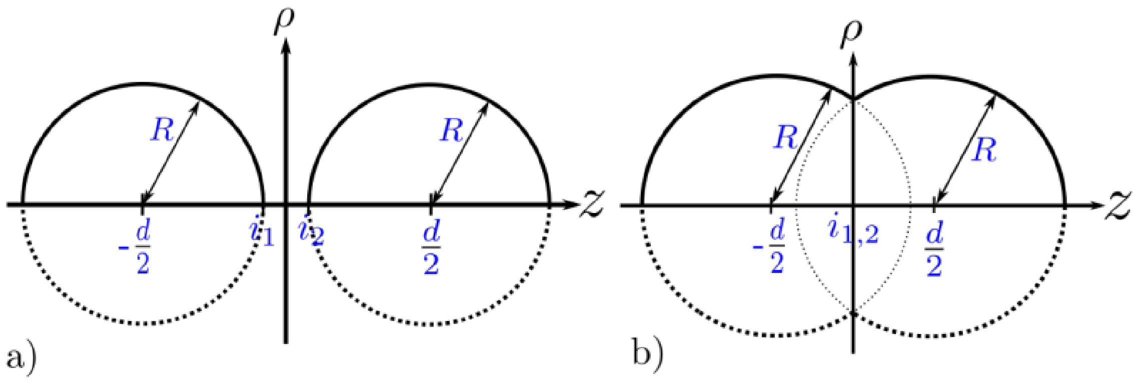

Supplementary Figure 14. QuantumPatch partition of space for V_C computation in cylindrical coordinates with ρ being the radial and z the axial coordinate. The host/dopant is considered to be at x=-/+ d/2. The union of the two spheres denotes the explicit space in which the polarization is taken into account by DFT simulations. The space outside the spheres is the implicit space which polarization is treated as a continuous medium with ϵ_r.

Supplementary Tables

Supplementary Table 1. Full names of the molecules.

Short name	Full name
TCNQ	Tetracyanoquinodimethane
F_4TCNQ	2,3,5,6-Tetrafluoro-7,7,8,8-tetracyanoquinodimethane
F_6TCNNQ	1,3,4,5,7,8-hexafluorotetracyanonaphthoquinodimethane
NPB	N,N′-Di(1-naphthyl)-N,N′-diphenyl-(1,1′-biphenyl)-4,4′-diamine
MeO-TPD	N,N,N',N'-Tetrakis(4-methoxyphenyl)benzidine
BF-DPB	N4,N4'-Bis(9,9-dimethyl-9H-fluoren-2-yl)-N4,N4'-diphenylbiphenyl-4,4'-diamine

Supplementary Table 2. Simulation parameters of molecules and undoped morphologies. The density is computed for undoped morphologies that can be compared to experimental data - not deviating over 12 %. The experimental intrinsic hole mobility is used to fit the disorder $\sigma_{IP,host}$ for BF-DPB and MeO-TPD (as explained in the manuscript section 4.3). The disorder of NPB is directly taken from literature. $\lambda_{hole/electron}$ denote the vacuum reorganization energies of holes and electrons, respectively, and ϵ_r the dielectric permittivity. Their computation is described in the manuscript, Section 4.3.

**Molecule**	**NPB**	**BF-DPB**	**MeO-TPD**
**Density of simulated morphologies,** g/cm^3	1.13	1.075	1.11
**Experimental density,** g/cm^3	1.14 [1]	1.21 [1]	1.2 [2]
**Experimental mobility,** $cm^2/(Vs)$	-	$5.7 \cdot 10^{-5}$ [3]	$2.3 \cdot 10^{-5}$ [3]
**Disorder of the host hole charge transport levels** $\sigma_{IP,host}$, **eV**	0.093 [4]	0.105	0.118
**Reorganization energy of electrons** $\lambda_{electron}$, **eV (vacuum)**	0.148	0.247	0.612
**Reorganization energy of holes** λ_{hole}, **eV (vacuum)**	0.282	0.207	0.211
**Relative dielectric permittivity** ϵ_r	2.730	3.267	3.100

Supplementary Table 3. Quadrupole moments of the host NPB (total charge = +1 e) and dopants (total charge = -1 e) based on TCNQ: TCNQ, F_4TCNQ, F_6TCNNQ molecules. See Supplementary Figure 1 for the orientation of the molecules relative to the coordinate system. Level of the theory: functional: PBE, basis set: def2-QZVP as implemented in Turbomole[6,7].

**Quadrupole tensor components** [(1)]	**NPB** (cation)	**TCNQ** (anion)	**F_4TCNQ** (anion)	**F_6TCNNQ** (anion)	**MeO-TPD** (cation)	**BF-DPB** (cation)
Q_{xx}	66.548038	-39.897168	-43.445547	-68.215376	92.382700	103.122000
Q_{yy}	-24.34399	8.338157	6.657296	17.235815	-23.579600	-58.72940
Q_{zz}	-42.20406	31.559011	36.78825	50.979561	-68.803200	-44.3930
Q_{xy}	-7.106605	-0.000074	-0.000629	-2.799074	9.237361	-38.379262
Q_{xz}	19.825208	0.000044	0.000078	-0.00015	3.649174	-0.067953
Q_{yz}	1.040408	0.000060	0.000092	-0.000067	-1.176883	-0.144202

[(1)] The unit of quadrupole tensor components is ea_0^2, e and a_0 being the elementary charge and Bohr radius, respectively.

Supplementary Table 4. The differences $IP_{host} - EA_{dopant}$ at low, medium and high dopant molar ratio (DMR). Low, medium and high DMRs means following. For BF-DPB:F_6TCNNQ: 0.0099, 0.0585, 0.3303; for MeO-TPD:F_6TCNNQ: 0.0040, 0,0554, 0.2269. “Low” and “high” were chosen as the smallest and highest DMR from experiments, while medium is the experimental value closest to 5%. Computed as described in the manuscript method Section 4.2 (IP/EA).

**DMR/Material**	**BF-DPB:F_6TCNNQ**	**MeO-TPD:F_6TCNNQ**
low	0.629 eV	0.540 eV
medium	0.675 eV	0.562 eV
high	0.841 eV	0.698 eV

Supplementary References

[1]: Nell, B., Ortstein, K., Boltalina, O. V. & Vandewal, K. Influence of Dopant–Host Energy Level Offset on Thermoelectric Properties of Doped Organic Semiconductors. *J. Phys. Chem. C* **122**, 11730–11735 (2018).
[2]: Li, J. *et al.* Measurement of Small Molecular Dopant F4TCNQ and $C_{60}F_{36}$ Diffusion in Organic Bilayer Architectures. *ACS Appl. Mater. Interfaces* **7**, 28420–28428 (2015).
[3]: Menke, T. *et al.* Highly efficient p-dopants in amorphous hosts. *Organic Electronics* **15**, 365–371 (2014).
[4]: Reiser, P. *et al.* Analyzing Dynamical Disorder for Charge Transport in Organic Semiconductors via Machine Learning. *J. Chem. Theory Comput.* **17**, 3750–3759 (2021).
[5]: Metz, W. D. Organic Crystals: Hints of Extraordinary Conductivity. *Science* **180**, 1041–1042 (1973).
[6]: Ahlrichs, R., Bär, M., Häser, M., Horn, H. & Kölmel, C. Electronic structure calculations on workstation computers: The program system turbomole. *Chemical Physics Letters* **162**, 165–169 (1989).
[7]: Weigend, F. & Ahlrichs, R. Balanced basis sets of split valence, triple zeta valence and quadruple zeta valence quality for H to Rn: Design and assessment of accuracy. *Phys. Chem. Chem. Phys.* **7**, 3297 (2005).
